# Importance of pre-mRNA splicing and its study tools in plants

**DOI:** 10.1007/s44307-024-00009-9

**Published:** 2024-02-08

**Authors:** Yue Liu, Sally Do, Henry Huynh, Jing-Xin Li, Ying-Gao Liu, Zhi-Yan Du, Mo-Xian Chen

**Affiliations:** 1 National Key Laboratory of Wheat Improvement, College of Life Science, Shandong Agricultural University, Taian, Shandong China; 2 National Key Laboratory of Green Pesticide, Key Laboratory of Green Pesticide and Agricultural Bioengineering, Ministry of Education, Center for Research and Development of Fine Chemicals, Guizhou University, Guiyang, China; 3 Department of Molecular Biosciences and Bioengineering, University of Hawaii at Manoa, Honolulu, HI 96822 USA

**Keywords:** Alternative splicing, Splicing machinery, Plant development, Stress response, Modern biotechnology

## Abstract

Alternative splicing (AS) significantly enriches the diversity of transcriptomes and proteomes, playing a pivotal role in the physiology and development of eukaryotic organisms. With the continuous advancement of high-throughput sequencing technologies, an increasing number of novel transcript isoforms, along with factors related to splicing and their associated functions, are being unveiled. In this review, we succinctly summarize and compare the different splicing mechanisms across prokaryotes and eukaryotes. Furthermore, we provide an extensive overview of the recent progress in various studies on AS covering different developmental stages in diverse plant species and in response to various abiotic stresses. Additionally, we discuss modern techniques for studying the functions and quantification of AS transcripts, as well as their protein products. By integrating genetic studies, quantitative methods, and high-throughput omics techniques, we can discover novel transcript isoforms and functional splicing factors, thereby enhancing our understanding of the roles of various splicing modes in different plant species.

## Introduction

Premature-mRNA (Pre-mRNA) splicing is an essential post-transcriptional regulatory process in eukaryotes. It has been extensively studied since the first discovery of RNA splicing by Richard Roberts and Phillip Sharp in 1977 (Berget et al. 1977). Currently, we know that eukaryotic genes consist of coding sequences (exons) and noncoding sequences (introns). Pre-mRNA alternative splicing involves the removal of various introns and non-coding mRNA segments, as well as the joining of corresponding coding sequences, resulting in the creation of different transcripts with varying combinations (Newman 1998; Gilbert 1978; Sharp 1987). Therefore, a single pre-mRNA can be processed into multiple mature transcripts through AS, thereby enhancing genetic diversity. AS can regulate protein expression by modifying the transcript to influence the stability or the ratio of specific isoforms (Wright et al. 2022). As a result, a multitude of proteins can be generated from finite pre-mRNA transcripts present in the cell, thus augmenting genomic and proteomic diversity (Panahi and Hejazi 2020). Given that AS plays a crucial role in gene expression, it holds particular significance in the development, stress response, and DNA damage response (DDR) for eukaryotes (Xie et al. 2023; Nimeth et al. 2020; Syed et al. 2015; Panahi and Hejazi 2020).

In plants, up to 90% of genes contributing to the proteome contain introns, and approximately 42% to 61% of genes undergo AS (Shang et al. 2017). Instead of primarily contributing to proteome diversity, AS plays a more significant role in enhancing the diversity of mRNA transcripts (Chaudhary et al. 2019). Additionally, in comparison to humans, the extent of AS contributing to plant proteome diversity is lower (Montes et al. 2019). It has been observed that similar to flowering plants, approximately 30% of pre-mRNA in *Chlamydomonas* undergo AS (Labadorf et al. 2010). As *Chlamydomonas reinhardtii* progresses through the cell cycle, splicing patterns vary across the phases (Pandey et al. 2020). However, this aspect was not investigated further, and the functionality of AS throughout the cell cycle phases remains unclear.

There have been numerous reports indicating that pre-mRNA splicing-induced changes in gene expression influence various aspects of plant development, as well as abiotic stress responses, including the circadian clock, flowering, flooding, and drought (Bao et al. 2023; Liu et al. 2016; Fávero Peixoto-Junior et al. 2018; Gil et al. 2017). Furthermore, several splicing factors have been confirmed using a combination of bioinformatics analysis and gene verification. This suggests that these factors can influence the splicing core components and signal pathways, thus impacting various plant biological processes (Xiong et al. 2019c; Liu et al. 2016; Fávero Peixoto-Junior et al. 2018; Cheng and Tu 2018). For example, *A.thaliana atu2af65b* mutant negatively regulates flowering due to a reduction in transcript abundance of the flowering repressor gene, *Flowering Locus C* (*FLC*). This results from increased IR and decreased transcript abundance of activators in *FLC*. In addition, light-activated photoreceptors regulate AS in *Physcomitrella patens* to modulate phototropic responses. These examples reveal that splicing is a crucial and intricate regulatory mechanism that needs to be understood in both eukaryotes and prokaryotes.

In animals, including humans, AS plays a role in functional mechanisms ranging from apoptosis to mRNA transcript concentration (Chaudhary et al. 2019). Therefore, several studies have revealed that uncontrolled AS can cause disease, and various investigations have been published to explore the use of AS in treating certain diseases, such as cancer (Chaudhary et al. 2019; Montes et al. 2019; Su et al. 2023). AS also increases genetic instability, which is heavily associated with tumorigenesis (Rahmutulla et al. 2014). AS is a key player in eukaryotic development. For example, it plays a role in determining the sex of *Drosophila* (Wright et al. 2022). Around 90–95% of human genes undergo AS (Chaudhary et al. 2019; Wright et al. 2022). However, most of these spliced genes are not translated into protein (Chaudhary et al. 2019).

Another significant impact of AS lies in its contribution to the phenotype. Although the process does not play as crucial a role as other transcription-related processes, it provides a rapid mechanism for the emergence of novel traits throughout evolutionary history (Wright et al. 2022). Thus, they have been attributed to the development of several phenotypic traits in pigmentation, muscles, and the nervous system in animals (Wright et al. 2022). As a result, AS can function in both regulating transcription and translation, contributing to the diversification of transcripts and, consequently, to the development of phenotypic traits (Lu et al. 2023; Wright et al. 2022).

Most splicing studies have primarily focused on land plants or crops, such as *A.thaliana* and rice (Marquez et al. 2012; Aghamirzaie et al. 2013). Additionally, there has been more progress in understanding AS types and events in higher plants (Bao et al. 2013; Gupta et al. 2015). However, the analysis of detected functional AS transcripts and the relationships among AS-induced isoforms has not been thoroughly explored.This review will profile relevant reports on AS regulation in plants, including the basic mechanism and differences in splicing systems between eukaryotes and prokaryotes, the functional analysis of splicing during development, and the responses to various stresses. Furthermore, several general and newly published strategies for the functional identification of alternatively spliced isoforms and transcript quantification are briefly discussed.

## Splicing mechanism and its molecular function in eukaryotic cells

### Basic mechanism of RNA splicing

RNA splicing, which involves the removal of introns within pre-mRNA, can be categorized into specific types. Firstly, there are cis-splicing and trans-splicing. The RNA produced through cis-splicing is created using exons located on the same pre-mRNA transcript (Eul and Patzel 2013). This process is favored over trans-splicing (Eul and Patzel 2013), which refers to the splicing of exons originating from different transcripts (Eul and Patzel 2013). There are also self-splicing introns that remove themselves from the pre-mRNA strand without the assistance of splicing machinery (Fica et al. 2013). Eukaryotic genes mainly undergo spliceosome–dependent splicing, which can be categorized into various splicing types, including cis-splicing (which encompasses classic and alternative splicing), trans-splicing (including SL trans-splicing and genetic splicing) (Simpson and Filipowicz 1996; Brown and Simpson 1998; Lorković et al. 2000; Blumenthal 2005).

When discussing the mechanism of mRNA splicing, the spliceosome remains an essential component. The spliceosome is a metalloenzyme that catalyzes the splicing of nuclear pre-mRNA (Hang et al. 2015; Will and Lührmann 2011). Currently, two types of spliceosomes have been recognized: the common major spliceosome that relies on U2 and the rarer U12-dependent spliceosome (Will and Lührmann 2011; Moyer et al. 2020). The former is composed of U1, U2, U5, U4/U6 snRNAs, along with various proteins to form small nuclear RNPs (snRNPs), while the latter is constructed from U11, U12, U5, U4atac/U6atac snRNAs, and their respective proteins(Will and Lührmann 2011; Vosseberg et al. 2022). Each snRNP plays a role in recognizing the pre-mRNA strand and identifying splice sites on the pre-mRNA, including the 5’ splice site (5’ss), 3’ splice site (3’ss), branch point sequence (BPS), and polypyrimidine tract (PPT) (Will and Lührmann 2011; Kováčová et al. 2020).

The mechanism of splicing has been extensively studied and is divided into two steps. In the first step, the 2’OH group of the conserved adenine in the BPS initiates a nucleophilic attack on the phosphorus of guanine located at the 5’ end of the intron (Hang et al. 2015; Will and Lührmann 2011). This process releases the 5’-exon and forms a lariat structure that resembles a lasso(Hang et al. 2015; Will and Lührmann 2011). Next, the 3’OH group at the 3’ end of the 5’ exon launches another nucleophilic attack on the phosphorus of the guanine at the 5’ end of the 3’ exon, resulting in the merging of the two exons and releases of the lariat structure (Hang et al. 2015; Will and Lührmann 2011).

However, for splicing to occur in some organisms, it is imperative that the spliceosome is assembled on the pre-mRNA. The first step of this process involves the U1 snRNP binding to the 5’ss, splicing factor 1 (SF1) binding to the BPS, and the U2 auxiliary factor (U2AF) binding to PPT (Will and Lührmann 2011). The pre-spliceosome or A complex is formed when the U2 snRNP binds with the BPS (Will and Lührmann 2011). Then, U4/U6 and U5 snRNPs are recruited to form the B complex (Will and Lührmann 2011). The destabilization of U1 and U4 snRNPs results in the formation of the final spliceosome (Will and Lührmann 2011). The catalytic activation by a helicase forms the B* complex, which can catalyze the first and second steps of splicing (Will and Lührmann 2011). Additionally, the formation of the C complex catalyzes the second splicing step (Will and Lührmann 2011). Ultimately, once splicing has occurred, the spliceosome can be disassembled and reassembled for splicing in another pre-mRNA (Will and Lührmann 2011).

In the splicing process, splice site recognition is crucial in determining the final RNA transcript. When splice site recognition, specifically intron definition (ID), incorrectly recognizes intron–exon boundaries, there is an increase in the occurrence of retained introns (RIs) (McGuire et al. 2008). When the exon definition (ED) process haphazardly identifies splice junctions, cassette exons (CEs) are produced (McGuire et al. 2008). At the conclusion of a study, researchers revealed that most eukaryotes can utilize both ID and ED, but animals prefer ED, whereas most fungi and protists prefer ID (McGuire et al. 2008). The latter results from the short introns found in fungi and protists that are more frequently identified by ID, due to a higher concentration of RI in these organisms (McGuire et al. 2008). For animals, given their long introns and shorter exons, CEs are more frequent, and they are more commonly identified by ED (McGuire et al. 2008).

There are many conserved components of the spliceosome in eukaryotes. For example, in *Porphyridium purpureum*, researchers have noted high IR, with half of the transcripts remaining unspliced (Wong et al. 2021). Generally, this species has low spliceosome functionality, possibly due to the reduction in accessory proteins and the unique U1 snRNP (Wong et al. 2021). More research on the spliceosome is necessary to better understand its function in splicing and how it can be influenced throughout the evolutionary timeline.

Splicing is also impacted by other factors. For instance, splicing can be increased by blocking the *transcription of ribosomal protein* genes (*RPGs*) (Munding et al. 2013). This suggests competition between pre-mRNA transcripts in the nucleus for processing machinery (Munding et al. 2013).

### Differences in splicing machinery between eukaryotic and prokaryotic organisms

A variety of characteristics have separated prokaryotic organisms from eukaryotic ones, varying from differences in intron type to spliceosome dependency. Introns are also classified into four categories based on their characteristics. These categories include self-splicing group I introns, self-splicing group II introns, spliceosomal introns, and archaeal introns (Haugen et al. 2005; Rogers 2019). Group I introns undergo self-splicing utilizing guanosine as a cofactor in its two-step process (Haugen et al. 2005). Additionally, they are found in a diverse group of eukaryotes, including algae and fungi (Haugen et al. 2005). Group II introns are more commonly found in mitochondrial and chloroplast genomes, as well as in bacteria (Haugen et al. 2005). Instead of guanosine, they utilize adenine within the intron to undergo self-splicing(Haugen et al. 2005). The small nuclear RNA (snRNA) that comprise the spliceosome originate from group II introns (Haugen et al. 2005). Group II introns utilize ribozymes or an RNA enzyme to hasten their removal from RNA transcripts (Fica et al. 2013). Spliceosomal introns are usually located in pre-mRNA found in the nucleus and employ a similar splicing mechanism as group II introns (Haugen et al. 2005). The last group of introns, tRNA/archaeal introns, are also located in the eukaryotic nucleus and RNA of Archaea (Haugen et al. 2005). These introns utilize an endonuclease and energy in the form of ATP to perform splicing, a process labeled as a “cut-and-rejoin mechanism” (Haugen et al. 2005).

Prokaryotes, especially bacteria, tend to harbor group I and II introns, although the frequency of these introns is lower than in eukaryotes (Nesbø and Doolittle 2003; LaRoche-Johnston et al. 2018). Group I introns are reported to be abundant in the genomes of mitochondria and chloroplasts (Nesbø and Doolittle 2003). For example, they have been found in some bacterial 23S rRNA genes (Nesbø and Doolittle 2003). Group I introns are not present in archaea, possibly because the tRNA splicing endonuclease and ligase system are more efficient in this domain (Tocchini-Valentini et al. 2011). These introns are only present in organisms that lack the aforementioned machinery (Tocchini-Valentini et al. 2011). Group II introns have been shown to serve other purposes that a ribozyme, and have been observed to increase RNA diversity through a trans-splicing mechanism (LaRoche-Johnston et al. 2018). This may also explain why they were conserved in bacteria, as increasing genetic diversity is thought to be beneficial for bacterial hosts (LaRoche-Johnston et al. 2018).

Splicing in prokaryotes is relatively uncommon due to the presence of polycistrons and their reliance on coupled transcription and translation (Lamolle and Musto 2018). They primarily possess ribozymes (Group I and Group II introns), which undergo self-splicing and trans-splicing to repair damage and expand proteomic diversity (Hausner et al. 2014; Cech 1990; Belfort 2017; Olson and Müller 2012). In contrast to prokaryotic mRNA, eukaryotic mRNA transcripts are monocistronic, containing genes composed of exons and introns. They undergo separate transcription and translation processes, which are advantageous for the evolution of complex splicing mechanisms (Keren et al. 2010). Many studies show that eukaryotic nuclear introns in protein-coding genes are derived from the invasion of group II introns. These introns or catalytic RNA with functions in self-splicing and transposable elements, are hypothesized to provide the framework for nuclear spliceosomal introns and other key components of the spliceosome (Sultan et al. 2016; Novikova and Belfort 2017; Rogozin et al. 2012; Valadkhan 2007; Mattick 1994). As a multi-megadalton molecule, spliceosome contains five snRNAs and over 200 different proteins. snRNAs are similar to self-splicing group II introns in both mechanism and structure, thus serving as the catalytic components of the spliceosome (Valadkhan 2007).

To date, most of the research on introns in prokaryotes has focused on introns in pre-tRNA. This is because mature tRNA plays an important role in translation, and some tRNA genescontain introns that need to be removed to form mature tRNA (Schwarz et al. 2020). Archaea and eukaryotic pre-tRNA have one or a few introns. However, the splicing of eukaryotic pre-tRNA introns does not lead to the synthesis of alternative transcripts (Hayashi et al. 2019). The introns found in archaeal and eukaryotic tRNA genes are spliced by proteins (Schwarz et al. 2020). This process is catalyzed by an endonuclease responsible for RNA splicing (Schwarz et al. 2020). The functional tRNA is produced by a tRNA ligase, which joins the spliced fragments together (Schwarz et al. 2020). Ribonucleases ensure the correct processing of pre-tRNA (Fujishima et al. 2011). In this process, endonucleases hasten intron splicing within the cell (Schwarz et al. 2020).In archaea, the tRNA splicing endonuclease has evolved to favor substrate specificity (Fujishima et al. 2011).

There are several major differences between eukaryotic splicing and prokaryotic splicing. Firstly, eukaryotes exhibit a greater variety of splicing types. They can undergo almost all types of splicing, including classical and alternative cis-splicing, genic and SL trans-splicing, as well as self-splicing, which significantly increases the diversity of the transcriptome and proteome (Bitar et al. 2013). Secondly, eukaryotic splicing can recognize splice sites more precisely with the help of snRNPs and additional proteins. In contrast, the prokaryotic self-splicing mechanism, which involves group II introns, lacks cofactors and depends on their elaborate 3D structure for excision (Vosseberg and Snel 2017; Borao et al. 2021). Lastly, eukaryotic spliceosome-dependent splicing is more complex than prokaryotic splicing. They involves multiple processes, such as the recognition of splice sites and branch points, the assembly and activation of spliceosome, the removal of introns, the ligation of exons, and the disassembly of spliceosome (Shi 2017).

### Current knowledge of splicing in the development and stress responses of plants

#### Alternative splicing in plants

RNA splicing is a fundamental mechanism in the regulation of multiple biological processes in plants under all environmental conditions. AS is a cis-splicing process that significantly contributes to transcriptome and proteome diversity by generating multi-transcripts from a single pre-mRNA (Yu et al. 2016; Harrison et al. 2002). Compared to classical cis-splicing, AS can recognize different splice sites to produce alternative transcripts from a single gene and consists of five categories: alternative 5’ splice site (Alt5’ss), alternative 3’ splice site (Alt3’ss), intron retention (IR), exon skipping (ES), and mutually exclusive exons (ME) (Keren et al. 2010). In plants, the most prevalent AS event is IR, which produces a mature transcript containing an intronic sequence. This occurs when the 5’ and 3’ splice sites of an intron are not properly recognized (Ner‐Gaon et al. 2004; Iida et al. 2004). If the unrecognized splice sites flank an exon, the exon will be removed along with the introns. Protein-coding mature transcripts created from IR are alternatively known as exitrons, and they may produce proteins with varying functionality (Marquez et al. 2015).

#### Role of splicing in the development of plants

The predominant form of RNA splicing in the mitochondria of angiosperms is Group II intron splicing. (Mower 2020). In mitochondria, the proper splicing of *NADH dehydrogenase subunit* (*nad*) is essential for the growth and development in *A. thaliana*, maize, and rice, including but not limited to seed development, embryo and endosperm development, pollen development, and the biogenesis of respiratory complex I (Zheng et al. 2021; Chen et al. 2017; Best et al. 2023; Wu et al. 2019b; Edris et al. 2023). Consistent with this, a study conducted has shown that when *nad7* is transformed into the nuclear genome, it successfully rescues the phenotype of the *slow growth3* mutant in *A.thaliana* (Hsieh et al. 2018). However, angiosperm mitochondrial introns are incapable of self-splicing (Bonen 2008); therefore, their splicing relies on numerous nuclear-encoded splicing factors. For example, the most common splicing factor in rice, maize and *A. thaliana* is pentatricopeptide repeat (PPR) protein. In maize, *Dek37*, *EMP603*, *EMPTY PERICARP16* are involved in the splicing of *nad1*(Dai et al. 2018; Fan et al. 2021; Xiu et al. 2016), while *PRP20*, *Dek35*, *ZmSMK9*, and *EMP32* are required for the splicing of *nad2*, *nad4*, *nad5*, and *nad7* respectively (Yang et al. 2020, 2021; Chen et al. 2017; Pan et al. 2019a). In addition, other nuclear-encoded proteins, such as chloroplast RNA splicing and ribosome maturation (CRM) protein, maturase (Mat) protein, and transcription termination factor, also act as splicing factors for *nad* (Lin et al. 2022; Chen et al. 2021c; Pan et al. 2019b). Similar to mitochondrial RNA splicing, the splicing of introns in chloroplasts requires an interaction with nuclear gene products. It has been discovered that PPR proteins can target not only mitochondria but also chloroplasts, thereby participating in the splicing of plastid genes (Huang et al. 2018). This interaction is vital for the biogenesis and development of chloroplasts. For instance, transcriptome analysis revealed that *EMB-7L* in maize participates in the splicing of various chloroplast genes, contributing to endosperm formation and overall plant development (Yuan et al. 2019). Additionally, certain PPR proteins in rice, such as *OsPPR6* and *OsPPR11*, are also indispensable for chloroplast splicing, which is crucial for proper chloroplast development (Zhang et al. 2023b; Tang et al. 2017). Apart from PPR proteins, other nuclear-encoded proteins like maturase, RNA helicases (RHs), and transcription termination factors also target chloroplasts and play a role in RNA splicing of chloroplast genes (Barthet et al. 2020; Gu et al. 2014; Hammani and Barkan 2014).

AS is a critical post-transcriptional step that regulates gene expression during developmental growth. One study found that the function of AS lies in the regulation of differentiation and control of developmental transitions (Li et al. 2016). In plants, 20% of multi-exon genes generate at least two isoforms through AS (Estrada et al. 2015). Additionally, in different tissues, there is preferential recruitment of polysomes to different functional isoforms (Palusa and Reddy 2015). Numerous studies reveal that AS in plants is closely associated with various developmental growth processes, such as the circadian clock (Zhang et al. 2023a; Liu et al. 2022a), root and embryo development (Xiong et al. 2019b, 2022; Lv et al. 2021), photomorphogenesis (Yan et al. 2022; Kathare et al. 2022), flowering (Gil et al. 2017; Qin et al. 2017; Xiong et al. 2019c; Lee et al. 2020, 2022; Chang et al. 2022; Yan et al. 2020), rice tillering (Liu et al. 2022b), fruit ripening (Sun and Xiao 2015), synthesis of beneficial compounds (Feng et al. 2022; Cai et al. 2022; Lawrinowitz et al. 2022) and senescence (Wang et al. 2021b) (Table 1). With the reduction in sequencing costs and the advancement of sequencing technology, an increasing number of plant species at various developmental stages of AS have become known. Transcriptome profiling has become one of the primary methods to investigate the potential roles of AS in specific plant tissues. In one study, it was found that 59.3% of multi-exon genes underwent AS in tomato tissues, including seedings, flowers, and young developing fruits (Sun and Xiao 2015). In the latter case, more transcript isoforms per gene were found, indicating that AS may serve more essential functions in the early-growth of fruits (Sun and Xiao 2015). The transcriptomic data of different tissues at different developmental stages from legumes, tea, radish, wheat, and hybrid poplar indicate that IR is the most common AS event (Iñiguez et al. 2017; Zhu et al. 2018; Luo et al. 2017; Yu et al. 2020; Wang et al. 2020; Chen et al. 2020a). However, in certain cases such as *G. hirsutum* and fleshy fruits, IR is not always the most frequent AS event (Zheng et al. 2023; Yan et al. 2021). This indicates that the alternative splicing patterns and splicing strategies are not always consistent across different developmental stages and species. Plants must regulate alternative splicing to achieve specific functions. In the moss *Physcomitrella patens*, it has been observed that light-regulated histone methylation leads to changes in AS patterns (Wang et al. 2021a). The analysis of DEGs, DAS genes, and DNA methylation in soybean nodule formation and cotton fiber development processes revealed that 40% and 23.31% of DAS genes exhibited differential expression in the respective processes (Niyikiza et al. 2020; Zheng et al. 2023). Additionally, increased DNA methylation was observed, suggesting that enhanced DNA methylation may contribute to the efficiency of AS.

**Table 1:** Summary of the fundamental studies on AS in plants during development and stress responses

Developmental growth				Stress response			
Plant species	AS detection	Growth scope	Ref	Plant species	AS detection	Stress type	Ref
*A. thaliana*	AS profiling; lincRNA	Root	(Li et al. 2016)	*A. thaliana*	Splicing Factor PRP31	Cold	(Du et al. 2015)
*A. thaliana*	AS profiling	Seeds	(Srinivasan et al. 2016)	*A. thaliana*	RNA-seq(IR)	drought	(Chong et al. 2019)
*A. thaliana*	AS profiling	Seed Dormancy	(Nakabayashi et al. 2015)	*A. thaliana*	PORCUPINE	Low temperature	(Capovilla et al. 2018)
*A. thaliana*	Splicing factor	Photomorphogenesis	(Xin et al. 2019)	*A. thaliana*	SR proteins	Abiotic	(Palusa and Reddy 2015)
*A. thaliana*	AtU2AF65b	Flowering	(Xiong et al. 2019c)	*A. thaliana*	AS profiling (IR)	Zinc tolerance	(Remy et al. 2014)
*A. thaliana*	AtBUD13	Embryo	(Xiong et al. 2019b)	*A. thaliana*	U1 snRNP Subunit LUC7	Salt; Cold	(de Francisco Amorim et al. 2018)
*A. thaliana*	AS profiling; AtSPF30	Circadian control	(Romanowski et al. 2020)	*A. thaliana*	full-length RNA-seq	Glucose Stress	(Du et al. 2022)
*A. thaliana*	AS profiling	Flowering	(Gil et al. 2017)	*A. thaliana*	RNA-seq; SmEb	Cold	(Wang et al. 2022)
*A. thaliana*	Splicing factor PRP18a	Roots; siliques	(Kanno et al. 2018a)	*A. thaliana*	RNA-seq; SmEb	Salt	(Hong et al. 2023)
*A. thaliana*	JANUS	Embryonic Pattern Formation	(Xiong et al. 2019a)	*A. thaliana*	SF1	Salt; Temperature	(Lee et al. 2020; Zhu et al. 2020)
*A. thaliana*	U11-48 K	Leaves; Infloresc-ence; Senescence	(Xu et al. 2016)	*A. thaliana*	CYP18-1	Heat	(Jo et al. 2022)
*A. thaliana*	PRP4KA	Pleiotropic phen-otype	(Kanno et al. 2018b)	*A. thaliana*	CYP18-2	Heat	(Lee et al. 2023)
*A. thaliana*	PacBio Iso-Seq	circadian	(Zhang et al. 2023a)	Rice	AS profiling	Cadmium stress	(He et al. 2015)
*A. thaliana*	XCT	circadian	(Liu et al. 2022a)	Rice	R2R3-MYB	Drought	(Fávero Peixoto-Junior et al. 2018)
*A. thaliana*	RES	root and embryo	(Xiong et al. 2022)	Rice	RS33	Salt and low-temperature stresses	(Butt et al. 2022)
*A. thaliana*	RDM16	Root stem	(Lv et al. 2021)	Barley	AS profiling	Low temperature	(Calixto et al. 2016)
*A. thaliana*	SWELLMAP 2	Seedling Photomorphogenesis	(Yan et al. 2022)	Maize	AS profiling	Drought	(Thatcher et al. 2016)
*A. thaliana*	SWAP1-SFPS-RRC1	Photomorphogenesis	(Kathare et al. 2022)	Soybean	AS profiling	Drought	(Song et al. 2020)
*A. thaliana*	AtSF1-FLM	Flowering	(Lee et al. 2022)	Soybean	AS profiling	Flooding; Drought	(Syed et al. 2015)
*A. thaliana*	RBP45d	Flowering	(Chang et al. 2022)	Tomato	RNA-Seq	Phosphorus	(Tian et al. 2021)
*A. thaliana*	Splicing factor1	Flowering	(Lee et al. 2020)	Tomato	AS profiling	Water; Nutrient	(Ruggiero et al. 2022)
*A. thaliana*	semi-qPCR	Pollen	(Estrada et al. 2015)	Linseed	AS profiling	Drought	(Wang et al. 2023b)
*A. thaliana*	Transcription factor BES1	Development	(Jiang et al. 2015)	Rose	RNA-Seq	Drought	(Li et al. 2020b)
*Brachypodium distachyon*	AS profiling	Flowering	(Qin et al. 2017)	*Beta vulgaris L*	RNA-Seq	Alkaline	(Zou et al. 2020)
Maize	RBM48	Endosperm	(Bai et al. 2019)	Cassava	AS profiling	Cold	(Li et al. 2020a)
Maize	Splicing factor	Endosperm	(Gault et al. 2017)				
Maize	Emp16	Seeds	(Xiu et al. 2016)				
Maize	AS profiling	Evolution	(Huang et al. 2015)				
Tomato	R2R3 MYB	Fruit ripening (pigmentation)	(Colanero et al. 2020)				
Tomato	RNA-seq analysis	Early growth fruits	(Sun and Xiao 2015)				
Watermelon	RNA-seq analysis	Seeds	(Saminathan et al. 2015)				

*Abbreviations*: *XCT* XAP5 CIRCADIAN TIMEKEEPER, *RES* retention and splicing

**Fig.1:**
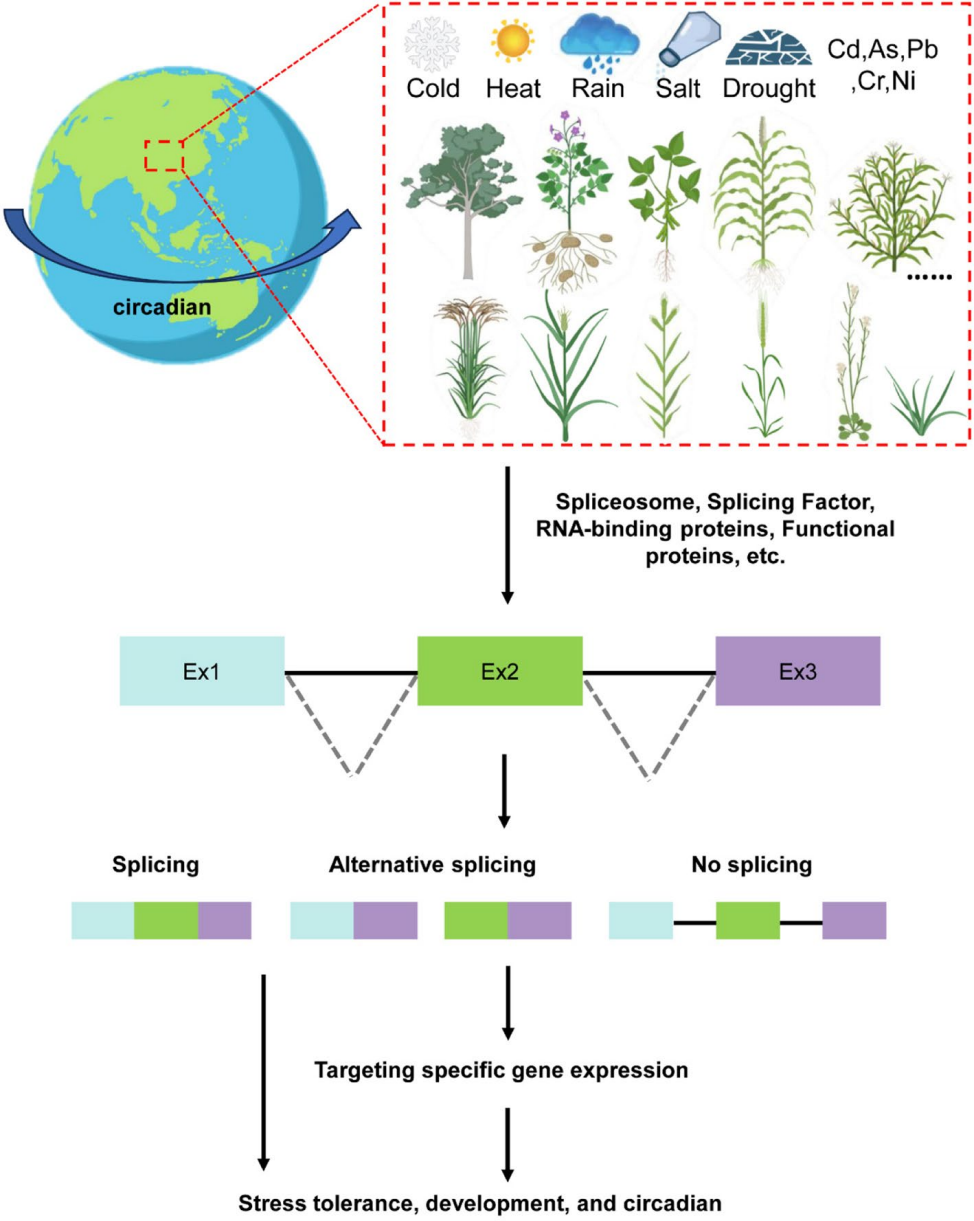
A possible AS regulation network exists for plant development, circadian rhythms, and stress response. Due to the Earth's rotation, all organisms are influenced by changes in light intensity. As a result, most species have developed an endogenous circadian clock that operates on a roughly 24-h cycle, known as the circadian rhythm. Splice-related factors, including spliceosome complexes, splicing factors, and functional proteins, target proper splicing of a serious of functional genes to regulate plant development, circadian and responses to abiotic stress. Different colored boxes represent exons and lines represent introns

There are several examples of developmental regulation mechanisms related to splicing in organisms (Fig. 1). Firstly, some genes can regulate plant development by producing different transcripts or affecting the ratio of different isoforms through AS. For instance, transcriptome data analysis has shown that certain NAC family (NO APICAL MERISTEM/*ARABIDOPSIS* TRANSCRIPTION ACTIVATION FACTOR/CUP-SHAPED COTYLEDONS) transcription factors (TFs) are involved in the regulation of leaf senescence during autumn. *PtRD26* is a NAC TF that produces a truncated protein, *PtRD26IR*, through IR. *PtRD26IR* can interact with hub senescence-associated NAC TFs in poplar, inhibit their DNA-binding ability and consequently exert a negative regulatory effect on senescence (Wang et al. 2021b). It is well known that the protein encoded by the *CONSTANS* (*CO*) gene can control photoperiodic flowering by activating rhythmic expression of the florigen, *flowering locus T* (*FT*). In *A. thaliana*, *CO* generates two protein isoforms (full-size *COα* and truncated *Coβ*), which results in abnormal flowering timing because *Coβ* lacks DNA-binding affinity and inhibits the stability of CO by affecting the interaction between Coα and E3 ligase (Gil et al. 2017). Thus, this is a common self-regulatory way for TFs, which generate transcripts with diverse DNA-binding abilities through AS. In *Brachypodium distachyon*, *FT2* produces two splice isoforms, *FT2a* and *FT2b*, by AS (Qin et al. 2017). The latter isoform affects the assembly of the flowering initiation complex, as the normal functions of *FT2a* and *FT1* are influenced by the *FT2b*-encoded protein, which forms heterodimers (Qin et al. 2017). A study found that two single-base mutations at the splice receptor site of *granule-bound starch synthase I* (*HvGBSSI*) caused abnormal RNA splicing and reduced amylose content in barley (Feng et al. 2022). Moreover, AS also regulates some critical genes associated with seed maturation and dormancy (Srinivasan et al. 2016; Nakabayashi et al. 2015). The delay in *germination 1* (*DOG1*) gene in *A. thaliana* generates five spliced variants, resulting in three protein isoforms. The regulation of seed dormancy by a single isoform necessitates the presence of the other two isoforms to avoid protein degradation (Nakabayashi et al. 2015). In conclusion, proper splicing is essential for development. Secondly, spliceosome complex contributes to the development of plants. For example, Pre-mRNA splicing can affect the growth of pollen tube. Spliceosome subunits, pre-mRNA processing factor 8, PRP8A and PRP8B, regulate the gene expression of pollen tube attractants *MYB98*, *CRP* and *LURE* in *A. thaliana* (Kulichová et al. 2020). The *A. thaliana* retention and splicing (RES) complex, consisting of *AtBUD13*, *GDS*, and *DDL*, counterparts of Bud site selection protein 13, U2 snRNP component Snu17, and Pre-mRNA leakage protein 1 in yeast, plays a regulatory role in root and early embryogenesis development by facilitating the splicing of multiple cell proliferation genes (Xiong et al. 2022). The spliceosomal component XCT (XAP5 circadian timekeeper) regulates the circadian clock, partly by controlling the AS of *LHY* (*LATE ELONGATED HYPOCOTYL*) and *TIC* (*TIME FOR COFFEE*) in *A. thaliana*. It associates with many SFs and is essential for 3' splice site recognition (Liu et al. 2022a; Zhang et al. 2023a). The U1 snRNP component RBP45d associates with the AS of *FLM*, thus regulating temperature-induced flowering (Chang et al. 2022). Therefore, spliceosome complexes or interactions between spliceosome complexes and functional proteins are essential for AS and plant development.

In addition, other factors, such as splicing factors, influence plant development through AS. One example is the loss of the splicing factor *ATU2AF65b* in *A. thaliana*, which can lead to the early-flowering phenotype by increased IR of the flowering repressor gene, *flowering locus C* (*FLC*) (Xiong et al. 2019c). Another example is an *A. thaliana* mutant of SF1, which reduces the functional *FLM-β* transcript level of a flowering time gene, *flowering locus M* (*FLM*), and increases the expression of another AS isoform, *FLM-δ* (Lee et al. 2020, 2022). Furthermore, the loss of the splicing factor PRP18a increases IR events and influences 3’ splice selection, resulting in plant phenotypes characterized by short roots and small siliques (Kanno et al. 2018a). Also, certain splicing factors, such as swellmap 2 and SWAP1-SFPS-RRC1 complex, physically interact with photoactivated phyB and regulate the AS of a subset of genes that play roles in both negative and positive regulation of photomorphogenesis in *A. thaliana*, respectively (Yan et al. 2022; Kathare et al. 2022). Therefore, SFs play a crucial role in regulating plant development through AS, primarily by increasing IR events that impact the generation of functional transcripts downstream.

In addition to spliceosomal complexes and splicing factors, certain functional proteins can also regulate plant development by influencing splicing. Homodimers formed by two RNA-binding proteins, KHZ1 and KHZ2, repress the splicing efficiency of *FLC* pre-mRNA and promote flowering in *A. thaliana* (Yan et al. 2020). The C2 H2 zinc-finger protein Du13 affects amylose content by regulating the AS of *Wxb* and other transcripts in rice endosperm (Cai et al. 2022). The Dwarf and High Tillering1 (DHT1), a monocotyledon-specific hnRNP-like protein, represses rice tillering by mediating the proper splicing of the strigolactones receptor gene *D14* (Liu et al. 2022b). Furthermore, AS is regulated by light to control photomorphogenesis. In the analysis of mRNA sequencing for AS changes in response to light in the moss species *Physcomitrella patens*, researchers discovered that many light-regulated AS transcripts and IR were induced upon exposure to light (Cheng and Tu 2018; Zhang et al. 2017a; Wu et al. 2014). In summary, AS of gene requires the regulation of multiple factors, which collectively form a network that facilitates normal growth and development in plants.

Additionally, there is evidence supports that AS is related to plant evolution. For example, the TF BRI1-EMS-suppressor 1 (BES1) responds to brassinosteroids (BRs) and has two isoforms: long *BES1-L* and short *BES1-S*. Compared to the common isoform *BES1-S*, *BES1-L* exhibits a more robust response to BR and uniquely exists in most *A. thaliana* ecotypes (Jiang et al. 2015). Additionally, a study examining the changes in AS within the transcriptomes of six teosinte and ten maize genera has revealed that greater complexity in AS levels is observed during the improvement from teosinte to maize. For example, there is a higher prevalence of transposable element (TE) sequences in maize, which may be tightly related to IR (Huang et al. 2015).

Finally, AS also plays a crucial role in biotechnology for the development of high-quality seedless fruits. AS changes have been observed in critical genes such as *Phloem protein 2*, *fructokinase-like 2*, and *nodulin MtN21* in both diploid and tetraploid watermelon, leading to the production of seedless crops (Saminathan et al. 2015). While an increasing number of AS genes and events have been uncovered in plants, further elucidation of the biological significance of AS genes in plant development is necessary.

#### Role of splicing in the stress responses of plants

Apart from its role in regulating plant growth and development, splicing also plays a crucial role in responding to various stresses (Fig. 1 and Table 1). Research has found that mitochondrial intron splicing, which depends on splicing factors OsNBL3 (PPR protein) in rice or CFM9 (CRM protein) in *A.thaliana*, enhances resistance to biotic and abiotic stresses (Qiu et al. 2021; Lee et al. 2019). Moreover, correct splicing of chloroplast introns mediated by chloroplast-targeting AtRH3 is essential for *A.thaliana* to resist salt and cold stress (Gu et al. 2014). Consistent with the above, the exploration of changes in intron splicing and expression patterns in chloroplasts of dicot (coffee) and monocot (rice) under different abiotic stresses (drought and temperature) revealed that it regulates the plant's response to abiotic stress (Dinh et al. 2016). Transcriptome analysis of maize (*Zea mays*) tissues, including the ear, tassel, leaf, and other tissues subjected to drought stress, reveals more than 48,000 novel transcript isoforms, with the majority of splicing changes occurring in the leaf and ear (Thatcher et al. 2016). Another study compared the genotypic variation of wild and cultivated tomatoes across a range of temperatures and show that modern varieties enhance short-term thermotolerance to high temperatures, which is associated with changes in splicing efficiency in response to heat stress (Hu et al. 2020). Furthermore, the analysis of transcriptome data from a rice diversity panel treated under both suitable and high salinity growth conditions reveals 764 significant genotype-specific splicing events that occur in saline conditions (Yu et al. 2021). In conclusion, splicing, especially AS, plays a key role in the response of plant to stress conditions. The following demonstrates how plants respond to various stresses in distinct manners.

##### Stress response regulated by different transcript isoforms

The impact of abiotic stress has been observed in rice, specifically in *Oryza sativa*. The abiotic conditions investigated in this study include drought, ABA, and salinity stress (Mukherjee et al. 2019). This study focuses on two isoforms of *galactinol synthase* (*GolS*), an enzyme that utilizes UDP-galactose and inositol to form galactinol (Mukherjee et al. 2019). Additionally, the expression of these genes is linked to stress responses, specifically drought and salinity stress (Mukherjee et al. 2019). The two isoforms of *GolS* in *O. sativa*, *OsGolS1* and *OsGolS2*, produce truncated *GolS*, a non-functional version of the enzyme. This leads to a different function of these genes as a method of controlling galactinol production (Mukherjee et al. 2019). Following this study, researchers determined that Regulated Unproductive Slicing and Translation (RUST) contributes to the stress response in *O. sativa* (Mukherjee et al. 2019). Uncovering this mechanism in rice’s stress response reveals the importance of splicing and may benefit future research on this topic in different species.

Another form of abiotic stress is high light. Under this condition, Liu B. et al. decided to observe *Cucumis sativus* seedlings, focusing on two transcripts of *Gibberellin 2-beta-dioxygenase* 8 (*CsGA2ox8*), and AS events at varying light intensities to determine their impact on the gibberellin (GA) hormone (Liu et al. 2021), which serves as a regulator for growth, specifically contributing to the hypocotyl height (Liu et al. 2021). The first transcript of the deactivating protein, *CsGA2ox8.1*, functions in the inactivation of GAs, whereas *CsGA2ox8.2* is not a functional isoform (Liu et al. 2021). Under high light condition, it was revealed that *CsGA2ox8.2* increases production to limit the levels of its functional counterpart (Liu et al. 2021). This would allow the organism to control its GA level. These results also suggest that AS has been used to alter the gene expression of *CsGA2ox8*. However, the mechanism by which this occurs is still unknown (Liu et al. 2021). This observation in *Cucumis sativus* may also be applicable to other plant species in terms of stress response, noting the importance of AS.

The TF *R2R3-MYB* gene in sugarcane (*ScMYBAS1*) has four transcript isoforms: *ScMYBAS1-2*, *ScMYBAS1-3*, *ScMYBAS1-4* and *ScMYBAS1- 5*. The higher expression abundance of two isoforms (*ScMYBAS1-2* and *ScMYBAS1-3*) in rice could contribute to higher relative water content, providing defense against drought stress (Fávero Peixoto-Junior et al. 2018). The splicing isoforms with IR in pre-mRNA coding for the glycine-rich proteins in *A. thaliana* are related to glucose stress (Du et al. 2022). In *A.thaliana*, the *zinc-induced facilitator 2* (*ZIF2*) gene encodes a transporter that regulates zinc (Zn) efflux and generates two transcript isoforms, *ZIF2.1* and *ZIF2.2*, by an IR event. The longer *ZIF2.2* splice variant exhibits greater Zn tolerance in comparison to *ZIF2.1* (Remy et al. 2014). The gene *NST1* (*NAC secondary wall enhancement factor 1*) of *Leucanthemella linearis* produces two transcripts with minimal difference, *LlNST1* and *LlNST1.1*, of which *LlNST1.1* responds to low temperature and salt stress. In conclusion, the study of functional differences in isoforms caused by AS is beneficial for establishing a material foundation for enhancing plant stress resistance (Wang et al. 2023a). Beyond the generation of AS isoforms, plants also specifically choose functional splice variants by preferential recruitment to respond to heat and cold stress (Palusa and Reddy 2015).

##### Stress response regulated by the spliceosome

To observe the stress response, *A. thaliana* was grown under extreme temperatures, osmotic stress, and salt stress, where three kinases were found to be essential in modifying spliceosome. All originate from *A. thaliana*: serine/arginine protein kinases (SRPK), *A. thaliana* FUS3 complement (AFC), and Pre-mRNA processing factor 4 (PRP4K) (Rodriguez Gallo et al. 2022). Throughout evolutionary history, previous studies suggest that independent replication events of SRPK have resulted in distinct versions of the kinase in different species (Zhang et al. 2020; Jia et al. 2023). However, the SRPK family itself remains conserved in eukaryotes and its function continues to regulate RNA editing (Rodriguez Gallo et al. 2022). Previous research on AFC reveals that the kinase continues to have important functions in plants (Rodriguez Gallo et al. 2022). Lastly, PRP4K is found to cluster in the *Brassicaceae* family due to recent replication events (Rodriguez Gallo et al. 2022). Therefore, this family of kinases plays an important role in RNA splicing especially for land plants (Rodriguez Gallo et al. 2022). To study the role of these proteins in AS and understand how they have persisted in photosynthetic eukaryotes throughout evolutionary time as a response to stress, Gallo M. C. R., et al. utilize phylogenetic analysis of the three protein families, as well as transcriptomic and bioinformatic analysis of *A. thaliana* (Rodriguez Gallo et al. 2022). As a result, this study revealed that the kinases studied in *A. thaliana* experienced changes in expression after exposure to cold, salt, and osmotic stress environments (Rodriguez Gallo et al. 2022). Light periods also had an effect on the expression in these kinases (Rodriguez Gallo et al. 2022). This study revealed the role of three kinase families in the abiotic stress response of *A. thaliana*.

In addition, spliceosome components are also closely related to plant stress fitness. 32 spliceosome-related proteins associated with stress granules were further studied to investigate the dynamic modifications of spliceosomal RNA-binding proteins under drought stress (Chen et al. 2021b). The spliceosomal core protein SmEb in *A. thaliana* regulates responses to chilling and salt stress by influencing AS events in different genes (Wang et al. 2022; Hong et al. 2023). Some proteins that interact with spliceosome components enhance stress tolerance by influencing the levels of AS isoforms. For example, *A. thaliana* spliceophilin cyclophilin 18–1 (CYP18-1), an accessory protein in the spliceosome complexes, interacts with splicing factors, U2 snRNA, and U5 snRNA, and is essential for IR in response to heat stress (Jo et al. 2022). Consistent with this is the CYP18-2, which interacts with U5 and U6 snRNAs to ensure that adequate levels of transcripts are present in response to heat stress (Lee et al. 2023).

##### Stress response regulated by SFs

Beyond that, splicing factors also play a role in regulating plant responses to stress through AS. For instance, in *A. thaliana*, the SF SKIP binds to the pre-mRNA of genes implicated in ABA signaling, thereby facilitating their precise splicing process and subsequent ABA-mediated AS (Zhang et al. 2022). Through gene expression and splicing analysis conducted on the rice *rs33* mutant under low temperature and salt treatment, it was discovered that RS33 (SR-rich SF) actively regulates the AS of several stress-response genes. As a result, it plays a crucial role in the rice plant's ability to respond effectively to abiotic stress (Butt et al. 2022). Furthermore, it was observed that *A.thaliana* sf1 mutant exhibit sensitivity to cold stress (Zhu et al. 2020; Lee et al. 2020). Transcriptomic analysis identified inadequate intron splicing efficiency in *atsf1* mutant, leading to abnormal expression of multiple genes (Zhu et al. 2020). Interestingly, the *atsf1* mutant exhibit a notable decrease in the abundance of the primary transcript of *FLM*, known as *FLM-β*, indicating a vital involvement of *AtSF1* in its temperature-dependent AS. This finding strongly suggests that *AtSF1* functions as a key regulator of temperature-responsive flowering (Lee et al. 2020).

##### Stress response regulated by AS events

Drought stress has also been investigated in *Rosa chinensis* to understand the adaptation method of the crop. A study published by Li W., et al. observed the plant’s leaves and roots in three different scenarios: no drought, mild drought, and severe drought (Li et al. 2020b). In response to this stress, the *R. chinensis*' genes between the roots and leaves of the plant underwent AS, where 42,544 isoforms were recorded (Li et al. 2020b). Additionally, ES increased in roses that were cultivated under drought stress compared to control conditions (Li et al. 2020b). This study revealed splice sites, newly discovered genes involved in the response to drought stress, and their regulatory mechanisms. These findings can contribute to future research on drought response in roses.

Generally, alkaline stress can impact the growth in plants, such as *Beta vulgaris* L. (Zou et al. 2020). Therefore, RNA-seq was utilized to observe transcriptional changes in *B. vulgaris* L. treated with no alkalinity and alkalinity for three and seven days (Zou et al. 2020). As a result, several genes undergoing differential alternative splicing (DAS) were observed (Zou et al. 2020). In the short-term treatment, eight genes underwent DAS, whereas in the long-term treatment, it was 16 (Zou et al. 2020). This underscores the importance of AS for *B. vulgaris* L.'s response to alkaline stress.

Similarly, AS also regulates how plants respond to cadmium (Cd) concentration by controlling splicing events. RNA sequencing of rice seedings under Cd stress reveals that post-transcriptional AS may regulate the response of plants to Cd stress (He et al. 2015). This is attributed to the identification of 542 unnormal splicing events from 476 differentially AS genes related to oxidation–reduction and chemical stimulus response (He et al. 2015). Interestingly, exitrons (exonic introns), a subset of exon-like introns identified in 2015 (Yu et al. 2016), are unusual AS events and widely effect protein function, as their splicing mediates stress response (Yu et al. 2016; Marquez et al. 2015). Therefore, future exploration in plant AS would deepen our understanding of their function.

Plants can also modulate the proportion of an AS event in response to stress. Transcriptome sequencing of cassava in response to cold stress revealed that IR is the most abundant AS type (Li et al. 2020a). In contrast, transcriptome sequencing analysis of soybean under drought treatment identified Alt3'ss and ES as the predominant AS types (Song et al. 2020). Therefore, the types of AS generated under different stress conditions may not always be consistent across different crops. In the *A. thaliana*, the BR-signaling kinase (BSK) family plays a crucial role in BR signaling. It was found that the rates of IR in *BSK5*, *BSK7*, and *BSK9* increased when *A. thaliana* was exposed to cold and heat stress (Li et al. 2019). These results suggest the function of BSK in response to temperature stress and may be valuable for future research on the stress response of specific tissues in *A. thaliana*. RNA sequencing of *A. thaliana* subjected to drought treatment indicates that more than 450 introns decreased IR, thereby improving splicing efficiency (Chong et al. 2019). The putative RNA helicase MOS4-associated complex 7 (MAC7) is closely related to normal IR events of spliced genes and is essential for plants’ defense against biotic and abiotic stresses (Jia et al. 2017). Therefore, the alteration of IR events has become an important strategy involved in improving plant tolerance to stress.

The methylation study on hybrid maize with different drought tolerance levels under water stress reveals varying levels of enhancement and a positive correlation between methylation levels and exon splicing events (Wang et al. 2021c). Similarly, another study on linseed suggests that under drought stress treatment, IR, Alt3'ss, and methylation levels increase significantly (Wang et al. 2023b). From this, it can be concluded that gene body methylation plays an important role in the regulation of certain AS events. However, a comprehensive analysis of the methylome and transcriptome of tomato seedlings under phosphorus deficiency revealed no enrichment of differentially methylated cytosines (DmCs) in differentially expressed genes (DEGs) and DAS genes (Tian et al. 2021). This could be attributed to the fact that the role of gene body methylation varies under different stress conditions.

##### Stress response regulated by evolutionary selection

The stress response mediated by AS appears to be connected to the evolutionary selection of plants. Circadian clocks are both evolutionarily innovative and essential for plants to anticipate and respond to day/night cycle and environmental fluctuations. A study has shown that many clock genes, such as *PRR3* in soybeans (*Glycine max*), respond to flooding and drought stress through a specific splicing pattern and other alterations (Syed et al. 2015). The changes in the paralogs of clock genes that respond to drought and flooding could be used to breed stress-tolerant varieties, promoting agricultural development (Syed et al. 2015). Similar to *A. thaliana*, clock genes in barley also respond to environment stress (such as low temperatures) through encoding different transcript isoforms. An example is the barley orthologue (*HvPPD-H1* gene) of *A. thaliana AtPRR7*. In addition, some conserved AS events of clock genes are observed between barley and *A. thaliana* (Calixto et al. 2016). This suggests that the same AS mechanism in response to environmental stimulus may exist in both dicots and monocots. Additionally, transcriptome studies of teosinte and maize have observed various AS level changes in genes related to stress responses during the evolution of maize (Huang et al. 2015).

## Modern biotechnologies used to study alternative spliced transcripts and their protein products

### Molecular cloning and genetic approaches

**Fig. 2:**
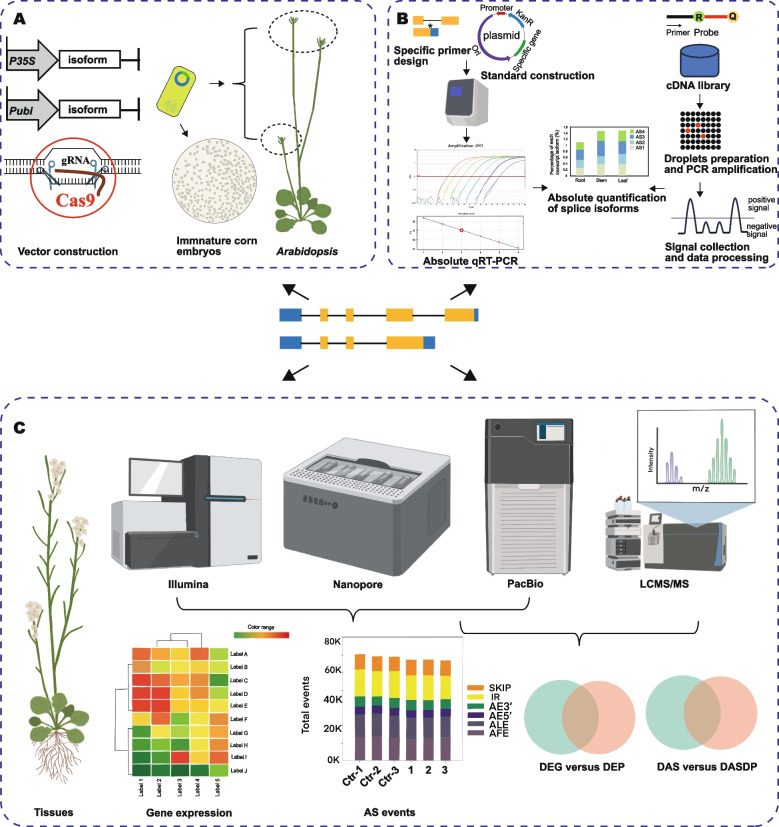
Representation of modern techniques for the study of alternative spliced transcripts and their protein products. **A** To understand the function of isoforms, obtaining the gene mutants with gain of function and knock-out is necessary. The genetic transformation of *A. thaliana* (representing dicotyledon) and maize (representing monocotyledon) are shown separately. Maize mutant lines require the co-culture of *Agrobacterium tumefaciens* carrying a target expression vector with immature corn embryos, followed by subsequent tissue culture. The corresponding *A. thaliana* mutant is created by infecting unfertilized inflorescence with *Agrobacterium tumefaciens* carrying target vector. P35S, CaMV 35S; Pubi, ubiquitin promoter. **B** QuantAS, an absolute quantitative method for splicing variants that combines qRT-PCR and dPCR. **C** Several high-throughput omics technologies. Spliced transcripts and their corresponding protein products can be obtained by combining second-generation transcriptome sequencing, represented by Illumina, with third-generation sequencing technologies, represented by Nanopore and PacBio, coupled with LC–MS/MS analysis. DEG, differentially expressed gene; DEP, differentially expressed protein; DAS, differentially expressed AS; DASDP, differentially expressed AS peptide

In plants, transgenic approaches are frequently employed for functional studies of spliced isoforms (Fig. 2A). Generally, vector systems for overexpression, RNA interference (RNAi), and CRISPR/Cas have to be constructed. For overexpression vectors, the coding sequence (CDS) of each spliced isoform is linked to strong promoters within cloning vectors. For example, the Cauliflower Mosaic Virus (CaMV) 35S promoter, which is commonly used in dicotyledonous plants, and the ubiquitin promoter, typically utilized in monocotyledonous plants (Ge et al. 2004; Kishi-Kaboshi et al. 2019). The RNAi vector system for RNA silencing can degrade specific sequences of homologous mRNA by recognizing double-stranded RNA as a signal (Guo et al. 2003). Beyond that, the CRISPR/Cas9 vector system for genome editing is simple and effective, and it has been widely used to delete target sequences or bases of spliced isoforms (Zhang et al. 2017b). To obtain different plant mutant lines, the successfully constructed vectors need to be transformed into *Agrobacterium tumefaciens*, which possesses a natural ability to infect injured areas of plants and induce crown gall formation (Van Eck et al. 2019). To date, *Agrobacterium tumefaciens* has been widely used to stably and transiently transform model plants and crops. For monocotyledonous plants, such as maize, corresponding mutants are usually obtained by transforming callus. For dicotyledonous plants, such as *A. thaliana*, *Agrobacterium tumefaciens*-mediated plant transformation is accomplished by infecting its inflorescence. The plant seeds collected after infection are screened for resistance and identified using PCR and real-time fluorescence quantitative PCR (qRT-PCR), and the available mutant lines are obtained through successive passage. Based on the phenotypes of different mutant lines, the biological function of the target spliced transcript can be determined. Furthermore, the function of the spliced isoform can be further verified by introducing the overexpression vector into knock-out mutant lines. In conclusion, molecular cloning and genetic approaches are essential for studying the function of specific genes or spliced transcripts in plants that can be gene-edited.

### Quantification of spliced isoforms

In the study of gene function, understanding the expression levels of different splice variants is crucial. Here, we summarize some techniques for quantifying splice isoforms (Table 2). Firstly, the conventional and commonly used method is semi-quantitative RT-PCR, which is easy to perform but does not distinguish variants with small sequence size differences (Riegler et al. 2021; Ferre 1992). In addition, real-time PCR has proven to be a reliable method for measuring the expression of spliced isoforms. It continuously monitors fluorescence signals, and the detected value can be counted as copy numbers using standard products with serial dilutions and standard curves (Vandenbroucke et al. 2001). Moreover, digital PCR (dPCR) is widely implemented for the relative quantitation of spliced variants due to its accuracy, high sensitivity, and reduced susceptibility to inhibitors (Van Heetvelde et al. 2017). Recently, a new method for absolute quantification of spliced isoforms, called QuantAS, has been developed (Song et al. 2023a) (Fig. 2B). QuantAS combines the respective advantages of qPCR and dPCR to achieve both amplification specificity and quantitative accuracy. To perform QuantAS, it is necessary to have access to known gene sequence, gene structure, and AS event information, as these are essential for designing specific primers and probes. Subsequently, the qPCR and dPCR data from the samples are analyzed and integrated to determine the copy number of the spliced isoform. The dPCR data is not affected by amplification efficiency because the instrument counts the number of positive droplets, represented by the fluorescence signal at the end of the reaction. Therefore, in comparison to qPCR, QuantAS can effectively reduce amplification biases. Furthermore, isoform classification relies on functional CDS, and QuantAS can streamline the workload by using distinct probes to simultaneously detect multiple isoforms simultaneously in a single reaction.

**Table 2:** Summary of quantitative methods of transcript isoforms in the last five years

Technique	Technique characteristic	Advantages and limitations	Refs
CircAST	RNA-seq reads were mapped to the genome, followed by the assembly and abundance estimation of alternatively spliced circRNA isoforms using multiple splice graphs and mathematical models	Extensive simulation and experimental validation showed that CircAST performs well in assembling full-length circular transcripts and quantifying exonic circular isoforms, even for transcripts with low abundance. But CircAST is applicable only to RNase RNase-treated samples and may miss intronic or intergenic circular isoforms.	(Wu et al. 2019a)
Label-Free Optical Biosensors	The method is performed when the target RNA sequence hybridizes with specific DNA oligos, the flanking RNA segments are degraded by RNase H enzyme	This method, using plasmon resonance biosensor, can quickly monitor AS of target gene in real time. Experiments aimed at detecting mRNA fragments within the pM − nM concentration range have shown an 81% accuracy when compared to RT-qPCR.	(Huertas et al. 2019)
HiFENS	HiFENS is an imaging tool for detecting endogenous isoforms based on hybridization chain reaction (HCR)	HiFENS is a high-throughput and high-specificity method that uses multiplexed HCR probes to quantitatively detect splicing isoforms in single cell. In comparison to traditional FISH, HiFENS is suitable for lower magnification objectives due to the signal amplification of HCR fluorescence. Importantly, HiFENS screens endogenous genes, thus reducing possible artifacts. However, HiFENS still has limitations in several aspects: it is challenging to detect rare isoforms with very short sequences; different targets require optimization in assay conditions; and new isoforms cannot be discovered due to the limitation of a pre-selected target.	(Shilo et al. 2022)
NanoCount	NanoCount is a transcript quantification tool designed for long-read direct RNA sequencing	Experimental results show that NanoCount can quickly and accurately quantify novel and known transcript isoforms, as well as identify differential gene and isoform expression.	(Gleeson et al. 2022)
ESPRESSO	ESPRESSO is a computational tool capable of discovering and quantifying spliced transcripts from error-prone long reads	When compared to StringTie2 and FLAMES, ESPRESSO exhibited superior performance in transcript discovery and quantification. It could discover new splice junctions and isoforms without relying on short-read RNA sequencing.	(Gao et al. 2023)
QuantAS	QuantAS can absolutely quantify AS isoforms by combining qPCR and dPCR	In comparison to traditional methods, QuantAS effectively mitigates the effect of amplification biases by combining qPCR (absolute quantification) and dPCR. It can concurrently identify multiple transcript isoforms in one reaction through multiplex PCRs employing different primers and their probes. However, QuantAS may face limitations when the similarity between two transcript isoforms is so high that specific primers cannot be designed.	(Song et al. 2023a)
Bambu	Bambu performs novel transcript discovery, allowing for the quantification of transcripts specific to the context from long-read RNA-seq data	Bambu enables more precise quantification of novel and known isoforms compared to most methods.	(Chen et al. 2023)
Mandalorion	Mandalorion is a tool to identify and quantify isoforms from full-length RNA-seq data	Real and simulated data processing analysis shows that the Mandalorion exhibits strong performance in terms of transcript identification precision and quantitative accuracy.	(Volden et al. 2023)

*Abbreviations*: *HiFENS* high throughput FISH detection of endogenous splicing isoforms, *FISH* fluorescence in situ hybridization, *HCR* hybridization chain reaction

### Multi-omics approach

Multi-omics approach can be used to study spliced isoforms, including the transcriptome by high-throughput sequencing and the proteome via mass spectrometry (MS) (Fig. 2C). Large-scale RNA sequencing from single cells can be applied to study the expression patterns of genes and isoforms across different cells (Sandberg 2014). Among the techniques for single-cell transcriptome sequencing, SMART-seq2 is one of the most commonly used and relatively mature methods that yield full-length cDNAs. It can analyze exons of each isoform and detect different alternatively spliced transcripts (Picelli et al. 2014). SMART-seq3 can detect a greater number of spliced isoforms with increased sensitivity (Hagemann-Jensen et al. 2020). Proteogenomics is a multi-omics approach that integrates proteomics with genomics and transcriptomics, and it is widely used in the study of AS (Chen et al. 2020b; Song et al. 2023b). For example, using srRNA-seq, lrRNA-seq, and liquid chromatography-tandem mass spectrometry/ mass spectrometry (LC–MS/MS) on rice seedlings under salt stress, researchers found 906,456 transcripts, of which 72.9% were spliced isoforms (Arefian et al. 2022). Comprehensive RNA-seq data analysis and Sequential Window Acquisition of All Theoretical Spectra (SWATH)-MS proteome data on poplar tissues under Pb stress were employed to focus on specific AS events (Chen et al. 2021a). It was further discovered that spliced isoform AS2 of the chaperone protein PtHSP70 has a strong affinity for lead and significantly increased gene expression under lead stress (Zhu et al. 2023).

## Conclusions and future perspectives

Splicing, as a crucial post-transcriptional regulatory mechanism, has been extensively studied. A comparison of eukaryotic and prokaryotic splicing mechanisms reveals that splicing in eukaryotes is more precise. The gene structure and gene expression characteristics of eukaryotes contribute to the evolution of complex splicing mechanisms. Recently, the advancement of sequencing platforms has dramatically promoted the progress of splicing-related research in various plant organisms. Many studies have found that different transcript isoforms resulting from AS events or base mutations at the splice site exhibit significant functional differences in development and stress response, highlighting the importance of proper splicing. Furthermore, an increasing number of splice-related factors, including spliceosome complexes, splicing factors, functional proteins, and light, have been demonstrated to significantly affect the correct splicing of many functional genes, thereby affecting plant phenotypes. Although the molecular mechanisms of several splicing-related factors have not been elucidated, their corresponding mutants have been shown to induce abnormal splicing of many genes. This further emphasizes the significance of proper splicing for plant development and stress response. To further uncover more AS regulatory mechanisms in plant species, genetic approaches combined with high-throughput sequencing are necessary for elucidating the role of transcript isoforms and for the discovery and quantification of novel transcripts. Accordingly, alternative isoform quantification methods have been developed to conduct experiments on both novel and known transcripts more conveniently and efficiently, which is significant for functional AS research.

## Data Availability

Not applicable.
